# Cultural influences and the Objective Structured Clinical Examination

**DOI:** 10.5116/ijme.5ff9.b817

**Published:** 2021-01-28

**Authors:** Reginald F. Baugh, Aaron D. Baugh

**Affiliations:** 1 Department of Surgery, University of Toledo College of Medicine and Life Sciences, Toledo, OH, USA; 2 Pulmonary, Critical Care, Allergy, Sleep Medicine, Department of Internal Medicine University of California San Francis-co Medical School, University of California San Francisco Medical Center, San Francisco, CA, USA

**Keywords:** Cultural influences, OSCE, USA

## Introduction

The objective structured clinical examination provides a credible scenario of patient interactionutilizing a standardized presentation that allows for student assessments. Like clinical medicine, objective structured clinical examinations are relational. Students speak with standardized patients in carefully structured, but dynamic interactions. Importantly, relational influences between standardized patient and students are operational,^1^^-^^3^ influencing student^1^^,^^3^ and standardized patient performance. Neither the impact of participants’ cultural backgrounds or attitudes nor their methods for overcoming their own unconscious biases are well-characterized.^4^ This research deficiency is important because an individual’s attitudes and beliefs influence their behavior.^5^ Individual motivation tempered by biographical experiences, learnings, cultural and institutional norms is influential when considering the effectiveness of communication in objective structured clinical examinations. In culturally discordant encounters, stereotype activation can influence decision-making,^6^ bias the information attended to,^7^ and affect the judgments reached^8^ regardless of whether performances (student or standardized patient) are stereotypical or not.^9^ Efforts to train away such differences have been largely ineffective.^10^^-^^13^ Collectively, student, standardized patient, and evaluator attitudes impact the range of learning objectives achievable.^14^

Culture is ingrained into the perspectives of all objective structured clinical examination participants with each culture producing different discourse rules: the meaning of words, gestures, interpretations, and reactions.^15^^,^^16^ What animates behavior in cross-cultural situations is not the same as that which motivates students where there is cultural concurrence. There are broad generalities such as a desire to be treated politely or receive honest explanations of medical issues. However, culturally specific expectations^17^ and presentations are important to cross-cultural clinical encounters. Even ostensibly universal humanistic values like empathy, altruism, respect, and accountability can have different cross-cultural manifestations.^18^^,^^19^ Different cultures can interpret and value the same presentation contrarily producing a valid assessment of competence of one group, but not another.^20^

Cultural values and implicit biases expressed through nonverbal cues can influence both the presentation choices standardized patients make and the ways students interpret and react to those choices.^21^^-^^23^Indeed, nonverbal communication is an important mediator of the positive effects of concordant clinical interactions^24^ as well as the perception of culturally competent communication.^25^ The maximal uptake of nonverbal signals^26^^,^^27^ requires awareness and understanding of cultural differences in expression^28^ neither standardized patients nor students uniformly possess. Consequently, standardized patients’ nonverbal behavior is often incongruent with student expectations.^29^^-^^33^ Ultimately, what communication cues the student does not receive or understand compromises the standardized patient-student interaction, empathetic student accuracy,^34^ and objective structured clinical examination outcomes. This is true independent of the student’s knowledge, skill, or earnestness. When cultural uncertainty is present as a result of inadequate communication, stereotypes are often substituted.^35^

The University of Toledo experience reflects the trade-offs that are to be expected with greater use of community-based diverse standardized patients. You may have to pay for them. The challenges related to health status and transportation are probably greater. Faculty prefer to teach to and for the perceived “average” patient: in the U.S. this means middle income to wealthy, well-educated non-Hispanic Whites. They argue that there is insufficient time to teach for multiple diverse populations. When feedback is obtained, at times, there is an attempt to discount the diverse perspective as not being representative. Some of the costs cited are real. Institutions should be realistic about the scale of the undertaking. However, these rationalizations for the perpetuation of cultural homogeneity ring hollow when compared to the benefits of reflecting the communities we serve. It misunderstands the larger trends shaping healthcare.

Europe, like America, is becoming more social and culturally diverse as a result of immigration and migration. There is also increasing recognition of how inequities in the care of more long-standing minorities internal to each country tax its healthcare system.  But medical education is largely unprepared for either, as neither class composition nor curriculum has evolved at pace with these demographic realities. Instructors are inadequately prepared, and the commitment of time and resources to address the acknowledged shortcomings of the system have been limited.^36^ It is time for medical education to adequately reflect the lived experiences of our local current and future patients, not the past. As part of a larger diversity and inclusion strategy, objective structured clinical examinations should be culturally aligned with the local context to account for different relationships and communication, hierarchical healthcare structures, conceptions of teams and interprofessional collaboration, and verbal/nonverbal communication practices. Majority interpretations may not be sufficient or applicable to understand marginalized populations’ interpretations of their own needs, perceptions, and experiences.^37^ Absent an effort to stay true to the local “real world” which is increasingly diverse, we undermine the fidelity of objective structured clinical examinations. While current guidelines for standardized patients acknowledge the potential for stereotyping, bias, and discrimination, they neglect this important element.^38^ Efforts to eradicate implicit bias with objective structured clinical examination training have been unsuccessful.^39^

Experiences that reflect each community’s diversity are best for learning or evaluation. Relational complexities inherent in outpatient or empathetic objective structured clinical examination narratives render standardized patient performances inferior compared to real patients.^32^^,^^37^ Moreover, standardized patient diversity may be pivotal for accurate student assessments in multicultural settings, and the overall satisfaction of future communication needs.^37^^,^^40^

When we do not account for the wide variety of cultural and religious frameworks patients will present with, students are left unprepared. It deprives the learner of an opportunity to discover and reflect on his or her reactions to such diversity. Interacting with diverse community standardized patients also reinforces the role that all patients are credible “experts” on the complexities of living with their condition in their community.^41^ They retain a unique perspective on student performance on objective structured clinical examinations not provided by students or instructor evaluations.^42^ Drawing on their knowledge and experience to inform their teaching, diverse standardized patients reflect not only the characteristics of the case, but the value systems related to the health and wellbeing of their cultural background, social group membership.^43^ In the absence of such a diverse community- and condition-specific participation, authenticity, as well as condition- and context-specific nuances are lost.

The potential positive contributions of diverse standardized patients and the recognition of the role cultural differences play in the delivery of quality healthcare is underappreciated. Addressing the multiple different cultures that can make up a community may be difficult. Further, multiple individual factors beyond culture, including experiences, mental models, and beliefs, may influence an objective structured clinical examination performance and its evaluation.^44^^,^^45^ However, there are tangible benefits to the fidelity of assessments and the quality of training for students. As medical schools work to make themselves more inclusive, culturally diverse standardized patients can tactically assist strategies to “fix the numbers, the institution, and knowledge”. ^46^ The fullest benefit of standardized patients will not be realized until we account for and accommodate the impact of culture more fully.

### Conflict of Interest

The authors declare that they have no conflict of interest.
